# A Prospect to Ameliorate Affective Symptoms and to Enhance Cognition in Long COVID Using Auricular Transcutaneous Vagus Nerve Stimulation

**DOI:** 10.3390/jcm12031198

**Published:** 2023-02-02

**Authors:** Lorenza S. Colzato, Julia Elmers, Christian Beste, Bernhard Hommel

**Affiliations:** 1 Cognitive Psychology, Faculty of Psychology, Shandong Normal University, Jinan 250014, China; 2 Cognitive Neurophysiology, Department of Child and Adolescent Psychiatry, Faculty of Medicine, Dresden University of Technology, 01307 Dresden, Germany; 3 Center of Clinical Neuroscience, Department of Neurology, University Hospital Carl Gustav Carus, Dresden University of Technology, 01307 Dresden, Germany

**Keywords:** long COVID, vagus nerve, atVNS, cognition, depression, brainstem, locus coeruleus, nucleus of the solitary tract, ecological momentary intervention

## Abstract

Long COVID, the postviral disorder caused by COVID-19, is expected to become one of the leading causes of disability in Europe. The cognitive consequences of long COVID have been described as “brain fog” and characterized by anxiety and depression, and by cognitive deficits. Long COVID is assumed to be a complex condition arising from multiple causes, including persistent brainstem dysfunction and disrupted vagal signaling. We recommend the potential application of auricular transcutaneous vagus nerve stimulation (atVNS) as an ADD-ON instrument to compensate for the cognitive decline and to ameliorate affective symptoms caused by long COVID. This technique enhances vagal signaling by directly activating the nuclei in the brainstem, which are hypoactive in long COVID to enhance mood and to promote attention, memory, and cognitive control—factors affected by long COVID. Considering that atVNS is a non-pharmacological intervention, its ADD-ON to standard pharmaceutical agents will be useful for non-responders, making of this method a suitable tool. Given that atVNS can be employed as an ecological momentary intervention (EMI), we outline the translational advantages of atVNS in the context of accelerating the cognitive and affective recovery from long COVID.

## 1. Introduction

Severe acute respiratory syndrome coronavirus 2 (COVID-19) is a coronavirus strain that produces COVID-19, the disease that is causing the current COVID-19 pandemic. It has led to over 6.5 million deaths and 620 million infections worldwide by October 2022. After 28 days, the majority of those who were infected (about 60%) appeared to have rid their bodies of the virus and can continue their regular activities [1]. Yet, about 30 to 40% developed long COVID, a postviral disorder caused by COVID-19 [1] that can affect people independent of age or the initial severity of the condition. “Long COVID” has been defined by a condition that occurs 3 months after the beginning of the infection, persists for at least 2 months, and cannot be accounted for by a different diagnosis [2]. People affected by long COVID experience “brain fog”, depression and anxiety, fatigue, breathlessness, cardiovascular complications, nausea, vomiting, abdominal pain, sleep disturbances, forgetfulness, and headache. As nicely pointed out by Orfei et al. [3], “brain fog” is a complex phenomenon, and to understand it, a biopsychosocial model is required that takes into account brain–body networks in interactions with psychological determinants. Such a framework will be crucial in the next years to make the correct diagnosis and to choose the appropriate treatment. Besides neurological symptoms, long COVID patients suffer also from cognitive deficits in the domains of attention, memory, and cognitive control, defined as top-down processes necessary for goal-directed thought and action, such as such as planning, decision making, and multitasking.

According to the WHO (https://COVID19.who.in accessed on 5 December 2022), in the first two years of the COVID-19 pandemic, at least 17 million people in Europe developed long COVID, suggesting that the consequences of this condition might become a leading cause of disability in Europe resulting in very high healthcare costs. New data in on health risks of reinfection show that, compared to individuals with first infection, reinfection causes adverse health outcomes, especially in unvaccinated people, increasing the likelihood of developing or exacerbating long COVID [4]. Considering that specific pharmaceutical agents to treat long COVID has not yet been developed, it is currently accepted to apply the so-called “treating the host” strategy using generic drugs. Accordingly, to evaluate treatments for long COVID, at least 26 randomized trials [5] are ongoing testing anti-inflammatory, antihistamine, antidepressant, anticlotting, and steroid medication. Given the well-known problem of non-responders in pharmacotherapy, it is important to develop potential novel ADD-ON neuromodulatory interventions to treat long COVID [6]. Furthermore, long COVID has been considered a complex condition likely to be triggered by multiple causes [5] and likely requiring a multidimensional approach taking care of the cognitive and affective, besides the neurological dimension. Hence, whereas current pharmacological interventions are mainly aimed to treat the neurological dimension, new ADD-ON tools might be particularly helpful to develop successful long-COVID treatments to ameliorate affective symptoms and to compensate for the cognitive failures caused by this condition. Restoring cognitive functions is particularly important, because preserved cognition is crucial for achievement in the educational and work environment [7], and an essential precondition for longevity in aging [8]. Furthermore, across the lifespan, intact cognition is considered to be a protective factor for mental resilience and mental health [9].

Several studies revealed long COVID to cause hypometabolism, especially in the brainstem, in both pediatric and adults patients [10,11,12,13]. Interestingly, the hypometabolism in the brainstem seems to correlate with the cognitive deficits caused by this condition [10,12]. In line with this evidence, a persistent brainstem dysfunction in long COVID has been hypothesized [14], suggesting that dysfunctional vagus nerve signaling [15,16], propagating from the brainstem to the brain might be an important neurological factor contributing to the cognitive deficits caused by long COVID. As support to this idea, impaired vagal activity has been reported in patients suffering from long COVID-19 [17].

Based on this hypothesis, we consider the feasibility of employing auricular transcutaneous vagus nerve stimulation (atVNS), a non-pharmacological, non-invasive brain stimulation technique [18] as an ADD-ON intervention to compensate for the cognitive failures caused by long COVID. AtVNS has several advantages over other methods that stimulate other branches of the vagus nerve, such as cervical transcutaneous vagus nerve stimulation (ctVNS). First, as pointed out by Karemaker [19], stimulating the cervical branch in the neck has the disadvantage to lack any specificity given that all nerves in the cervical bundle (the lesser occipital, great auricular, transverse cervical, supraclavicular, ansa cervicalis, phrenic nerve, nerve to rhomboids, and nerve to serratus anterior) are targeted indistinctively producing potential adverse effects which can limit the administration of ctVNS. Hence, compared to ctVNS, atVNS is very specific because it uniquely targets the auricular branch of the vagus nerve and no other cranial nerves [18,20,21]. Second, atVNS has been proven to enhance vagus nerve signaling by directly activating the nuclei in the brainstem, such as the nucleus of the solitary tract (NST) and the locus coeruleus (LC) [20,21,22,23,24,25,26], which are known to be hypoactive in individuals suffering from long COVID [10,11,12,13]. In contrast, several imaging studies carried out in the last three years using ctVNS [27,28,29,30,31] did not reveal any direct activation of the NST and the LC. Third, atVNS has been demonstrated to successfully enhance vagal tone, as measured by HRV [32,33,34], which has been found to be significantly low in long COVID patients [17]. Fourth, quantitative evidence, as supported by meta-analytical studies, showed that atVNS can reliably ameliorate symptoms in depressive patients [35], and can enhance attention, memory, and cognitive control [36]. Note that these are the same processes impacted by long COVID [37], which are known to be driven by catecholamines and GABA, neuromodulators synthesized by the same nuclei in the brainstem impacted by long COVID. In contrast, even though ctVNS has been found to preliminarily enhance psychomotor vigilance in sleep deprived individuals [38] and to be quantitatively effective in relieving acute pain for migraine and cluster headache [39], to date, there is no quantitative evidence that ctVNS is effective in the treatment of depression or in enhancing attention, memory, and cognitive control. Accordingly, we believe that, given that ctVNS has already obtained FDA clearance for the treatment of headache (but not specifically for COVID-19 patients) and of asthma caused by COVID-19 (https://www.fda.gov/news-events/press-announcements/coronavirus-COVID-19-update-daily-roundup-july-13-2020 accessed on 5 December 2022), it might potentially be suited for the treatment of these specific neurological symptoms caused by long COVID, whereas atVNS is more indicated to ameliorate affective symptoms and to enhance cognition in long COVID, the main factors targeted by this article.

In the following, we describe the involvement of the brainstem and vagus nerve signaling in long COVID and discuss the neuromodulator importance of nuclei in the brainstem (such as the NST and the LC) in attention, memory, cognitive control, and for affective processes. Next, we will examine the cognitive deficits induced by long COVID. Finally, we will consider the mechanism of action of atVNS, followed by proposing atVNS as a feasible ADD-ON intervention, with various translational advantages. Because of the wearability of the ear electrode and at-home self-administration without medical supervision, atVNS can be employed as an ecological momentary intervention (EMI) and be very useful in everyday life in the context of accelerating the affective and cognitive recovery from this post-viral disease.

## 2. The Role of Brainstem and Vagus Nerve Signaling in Long COVID, Affective Disorders and Cognition

Long COVID has been considered a complex condition likely to be triggered by multiple causes [5]. Hence, to understand the causes of long COVID, it is crucial to develop different approaches and therapies to treat this condition. Several hypotheses, not mutually exclusive, have been developed in the last two years to account for long COVID, ranging from viral persistence, which would result in an excessive production of proinflammatory cytokines [40], to autoimmunity, suggesting that antibodies generated by COVID-19 erroneously assault the body’s own proteins [41], or to persistent micro-clots generated by the infection that would prevent oxygen flow to tissues [42]. Another intriguing hypothesis put forward, and on which we will focus in this article, is that long COVID might be due to a persistent brainstem dysfunction [14]. According to this hypothesis, COVID-19 may attack the brainstem via direct viral assault. The virus is known to infect the cells through the binding of spike proteins to angiotensin-converting enzyme 2 (ACE 2) and neuropilin-1 receptors, which are highly expressed in the brainstem [43]. High expression of these receptors in the brainstem might promote the COVID-19 infection in this specific brain area, causing long-lasting damage [14]. This idea is supported by brain autopsies, which have revealed severe neurodegeneration in the brainstem [44,45], and by imaging evidence [10,11,12,13]. Indeed, several imaging studies revealed long COVID to cause hypometabolism, as measured by 18F-FDG brain PET, especially in the brainstem, in both pediatric and adults patients [10,11,12,13]. Interestingly, the hypometabolism in the medulla (where the NST is located) and in the pons (where the LC is situated) of the brainstem seems to correlate with the cognitive deficits, such as executive and memory impairments, caused by this condition [10,12]. Furthermore, the brainstem noradrenergic systems seem to be involved in the pathogenesis of anxiety and depression [46], which are two of the main affective symptoms reported in long COVID [2].

A second route by which the virus may affect the brainstem is via the blood–brain barrier and via trans-synaptic transfer of the virus through the glossopharyngeal nerve, which is tightly connected to the branches of the vagus nerve [14]. Consistent with this idea, a disrupted vagus nerve signaling has been proposed as an important neurological factor contributing to long COVID [15,16]. Indeed, as pointed out by Proal and VanElzakker [15], the sensory vagus is characterized by receptors able to detect proinflammatory cytokines. When this happens, a neuroimmune response is activated, triggering a so-called “involuntary sickness response” encompassing symptoms like fatigue, nausea, fever, and stomachache. A prolonged and continuous production of proinflammatory cytokines attacking the vagus nerve can cause an enduring involuntary sickness response associated with chronic symptoms, such as in long COVID. Consistent with this hypothesis, a recent study by Acanfora et al. [17] found evidence of impaired vagal activity as indexed by low heart rate variability (HRV), which is a biomarker of vagal tone [47]. Notably, long-COVID symptoms and vagal functioning share a significant amount of similarities. Abnormal vagus nerve functioning seems to be involved in affective disorders, such as depression, as indicated by altered frontal–vagal network in the brain of people suffering from this disorder [48]. Indeed, meta-analytic evidence demonstrated the effectiveness of enhancing vagal signaling in ameliorating depressive symptoms [35]. It therefore makes sense to assume that an altered function of the vagus nerve might contribute to the affective symptoms reported in long COVID. Furthermore, vagal efferent fibres are innervating the heart, the lungs, and the gastrointestinal tract, and are responsible for the circadian rhythms and correct cardiac, respiratory, and digestive functioning [49]. Not surprisingly, likely because of dysfunctional efferent vagal signals, long-COVID patients suffer from neurological symptoms exactly in those domains: sleep disturbances, cardiovascular complications, breathlessness, nausea, vomiting, and abdominal pain. The vagus nerve does not only entail efferent but also afferent fibres to the brain [50]. The afferent vagal signal is transmitted from peripheral nerves to nuclei in the brainstem, such as the NST and LC, before reaching the hippocampus and cortical areas, including the insula, the prefrontal cortex (PFC), and the motor cortex [18]. The NST and LC synthesize GABA and noradrenaline (NA), two main neuromodulators known to be involved in many cognitive processes, including attention, memory, and cognitive control (i.e., planning, decision making, and multitasking) [51,52,53]. Specifically, GABA plays a crucial role in control processes [54,55], such as response inhibition and conflict monitoring [56,57], whereas NA seems to be involved in supporting response selection processes and task-related decisions [58,59,60,61]. Interestingly, as we will see in Section 3, long COVID patients suffer from deficits exactly on those cognitive domains, suggesting a disrupted afferent vagal signaling in this condition.

In sum, dysfunctional efferent vagal signaling can account for neurological symptoms, whereas dysfunctional afferent vagus nerve signaling, propagating from the brainstem to the brain, might be an important neurological factor contributing to the affective symptoms and cognitive deficits caused by long COVID.

## 3. Cognitive Deficits in Long COVID

Deficits in attention, memory, and cognitive control processes characterize both acute infection [62] and long COVID [37,63]. Regarding long COVID, meta-analytical evidence based on 43 studies showed that at least 12 weeks after confirmed COVID-19 infection 32% and 22% of the patients (*n* = 13,232) suffered from fatigue and cognitive deficits, respectively [62]. Interestingly, it seems like that worse cognitive performance is more frequent among women and is associated with the severity of neuropsychiatric symptoms, sleep disorders, and rumination index [3]. Cognitive deficits in the domains of attention, memory, and cognitive control processes, such as planning, decision making, and multitasking were systematically reported. First, long COVID patients consistently described experiencing attentional dysfunctions, especially in terms of sustained and focused attention [64,65,66,67,68,69,70]. These self-reports were confirmed by objective measurements, as indexed by neurocognitive assessment batteries, indicating poor performance in the domain of attention and information processing [71,72]. Second, along the same line, long COVID patients reported short-term memory loss [64,65,66,67,68,73,74,75,76,77] and these subjective self-reports were also supported by objective assessments [71,72]. Third, among long COVID patients, cognitive control seems also strongly impaired, especially in terms of multitasking, cognitive flexibility, planning, and interference control [78,79,80,81,82,83]. These results should not be surprising, given that attention, memory, and cognitive control are known to be modulated by GABA and catecholamines [54,56,57,58,59,60,61], which are synthesized by the NST and the LC—the nuclei severely impacted by long COVID [10,11,12,13].

In sum, long COVID patients seem to suffer from deficits in attention, memory, and cognitive control, processes driven by GABA and catecholamines, neurotransmitters originating from the NST and the LC located in the brainstem—which is damaged by long COVID. However, it is important to keep in mind that most of the studies described above were observational and do not investigate the neurobiological underpinnings underlying these deficits. From these kinds of studies, it is also not possible to infer any causal relationships, as it was not, for instance, ascertained whether the reported deficits were present preceding the COVID-19 infection. Further, it is not possible to exclude that cognitive impairments were the mere consequence of fatigue, given that fatigue has been found to cause cognitive deficits [84]. However, a recent study has pointed that in the case of long COVID, fatigue and cognitive deficits co-exist, but are two independent and distinct sequelae of COVID-19 infection [85]. Finally, as pointed out by Orfei [3], in order to make the correct diagnosis of cognitive deficits in long COVID and to choose the appropriate treatment, it will be necessary to adopt a biopsychosocial model including psychological factors.

## 4. Clinical Studies Employing atVNS

AtVNS is a U.S. Food and Drug Administration (FDA) recognized intervention used in clinical settings to treat disorders such as pharmacoresistant depression and epilepsy [86]. Regarding depression, a sham-controlled pilot study in depressive patients revealed that two weeks of atVNS intervention was enough to ameliorate depression severity indexes [87]. These pilot results have been confirmed by another study with a bigger sample size [88]. Neuroimaging evidence in depressive patients showed that atVNS modulated functional brain connectivity in the default mode network [89,90] and caused insula activations that were predictive of the clinical efficacy of tVNS treatment [91]. Indeed, meta-analytical evidence on depression showed that enhancing vagal signaling this novel treatment, compared to sham stimulation, can reliably normalize the frontal–vagal network in the brain and improve depressive symptoms—thus providing a non-pharmacological alternative tool to treat depression [35].

Concerning epilepsy, a pilot study revealed that after nine months of intervention, seizure frequency declined in five out of seven patients [92]. The findings were replicated in another study in children reporting reduced seizure frequency as a consequence of an intervention of six months [93]. Consistent with this picture, a sham-controlled study in 27 patients suffering from epilepsy found reduced seizure frequency after an intervention of five months [94]. However, it is important to keep in mind that a large-scale clinical trial of atVNS in epilepsy is pending and more data are needed to evaluate the real efficacy of atVNS for epilepsy [95].

Besides depression and epilepsy, atVNS has been successfully applied in the management of tinnitus. A pilot study revealed that an intervention of 10 days of atVNS in combination with sound therapy was enough to reduce patient-reported tinnitus severity [96]. On the same line, subsequent studies found similar results when atVNS was combined with sound therapy [97,98] but not when atVNS was administered alone [99]. Further, atVNS has been successfully administered to compensate for cognitive decline in aging: compared to sham stimulation, active atVNS enhanced the number of hits in an associative face–name memory task in an elderly sample [100].

In sum, even if more converging evidence is necessary, the studies described above indicate that atVNS has the potential to ameliorate a wide range of clinical conditions, such as depression, epilepsy, and tinnitus.

## 5. Neurobiological, Affective and Cognitive Effects of atVNS

**Figure 1:**
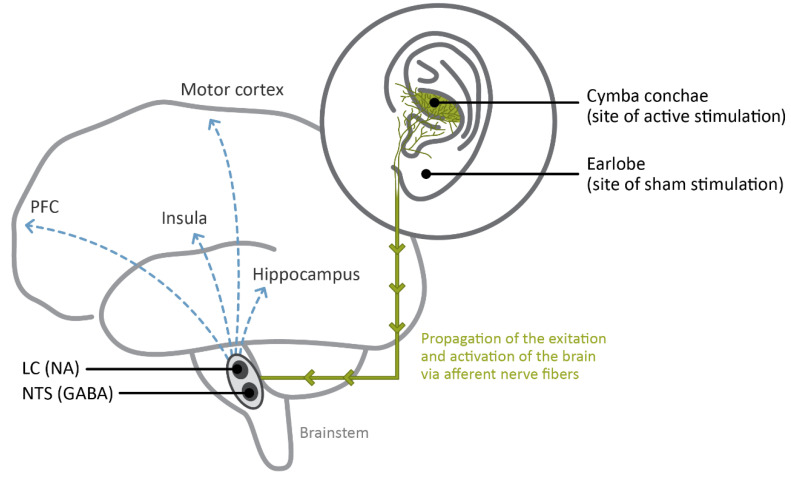
Graphic sketch of the main brain areas affected by atVNS: the NST and the LC in the brainstem, which synthetize GABA and NA. In a bottom-up fashion, the activation spreads from the auricular branch of the vagus nerve located in the cymba conchae to these nuclei and from there to higher subcortical and cortical areas, such as the hippocampus, the insula, the PFC, and the motor cortex. On the right, the illustration of the ear indicates the correct placement of atVNS electrodes for active stimulation (via the cymba conchae) and for sham stimulation (via the earlobe, which is known to be free from vagal innervation), as frequently used in experimental settings.

As pointed out by Colzato and Beste [18], the vagus nerve fibers in the brainstem terminate in different brain structures: the NST and the spinal trigeminal nucleus which include vagal afferent fibers, and the nucleus ambiguous and dorsal nucleus of the vagus, which contain vagal efferent fibers [101,102]. AtVNS activates the vagus nerve by means of a specific earplug electrode designed to fit the pinna of the external (outer) ear, where the auricular branch of the vagus nerve is positioned [18,103,104]. From the mounted earplug electrode, the stimulation propagates bottom-up from the afferent fibers to the brainstem (i.e., where the NST and LC are positioned) to the hippocampus, the insula, the motor cortex, and the PFC [18] (see Figure 1).

Compared to cervical tVNS and invasive VNS, which stimulate both efferent and afferent vagal fibers, atVNS stimulates only afferent fibers to the brain [50]. A large body of imaging literature shows that atVNS activates the brainstem, specifically the NST and the LC [20,21,22,23,24,25,26]. This is noteworthy and of crucial importance, given that those nuclei in the brainstem have been found to be hypoactive in long COVID patients [10,11,12,13]. Several neurotransmitter systems are assumed to be impacted by atVNS [105]. However, the main GABAergic and noradrenergic pathways in the brain arise from the NST and the LC, and the primary neuromodulators being affected are GABA and NA [106,107]. Interestingly, the NST afferent fibers mainly project to the LC making of the NST-LC structures the main target of activation by vagus nerve stimulation [108]. Accordingly, as proposed by Konjusha et al. [105], the selectivity of activation in those areas makes of atVNS a better suited tool to release GABA and NA than pharmacological agents acting on the GABAergic and noradrenergic systems [104]. As GABA and NA are involved in many cognitive processes, including attention, memory, and cognitive control (e.g., planning, decision making, and multitasking) [51,52,53], it is not surprising that literature reviews and meta-analysis found atVNS to be an effective tool to promote exactly those cognitive functions that are also deficient in long COVID [18,36].

As suggested by Colzato and Beste [18], the choice of stimulation parameters (i.e., stimulation frequency, pulse width, on-off cycle, current intensity, location of stimulation, side of stimulation, position of sham stimulation, and time of stimulation) of atVNS are crucial to attain an optimal treatment of depressive symptoms and an enhancement of cognitive functions. So far, most of the studies examining the effects of atVNS in humans are employing commercially available equipment, such as NEMOS^®^, with settled parameters such as current intensities till 5 mA, frequency of 25 Hz, 200 μs pulse width, and 30 s on/30 s off-cycle or continuous stimulation. Despite the fact that several studies have demonstrated that right atVNS does not induce cardiac side effects [32,33,34], atVNS is usually administered to the left ear given that the left vagus has no projection to the heart, hence, preventing and limiting any possible cardiac adverse reactions [18]. Regarding the location of stimulation, an imaging study has extensively examined several vagally innervated areas of the ear showing that the strongest activation of the NST and the LC has been reached by stimulating the cymba conchae [20]. Concerning the sham stimulation, the earlobe has been regarded as the most suited position given that it has been demonstrated to be clear from vagal innervation [109]. Indeed, the stimulation of the earlobe has been shown to not cause any activation of the NST and the LC [20] as proof that this location does not activate vagal afferents in the brain.

With respect to current intensity, stimulation frequency, and pulse width, animal literature (rats) using invasive VNS showed that higher stimulation frequencies (120 Hz) trigger the maximal firing rates of the LC [110]. Interestingly, this finding was recently replicated in humans using atVNS [111]. Short bursts of atVNS increased evoked pupil dilation (i.e., index of noradrenergic activity) in a positive fashion in response to stimulation parameters: higher stimulation settings (pulse width *x* intensity) were associated with enhanced pupil dilation, suggesting maximal activation of the LC [111]. However, the positive correlation between stimulation intensity and LC activation was not accompanied by best cognitive performance in human patients suffering from epilepsy [112]. Indeed, only intermediate stimulation intensities were linked to optimal performance in a task measuring recognition memory [112], as indication that stimulation intensity and cognitive performance might follow an inverted U-shaped function: intermediate stimulation, but not high or low intensities, might trigger the ideal levels of GABA and NA release which is known to support the best cognitive performance [58].

Regarding the amount of time of stimulation, a recent feasibility study in depressive patients suggested that a four week intervention, with four hours stimulation daily, was enough to induce a significant decrement in depression severity and an increment in cognitive speed [113]. When considering all the studies described above and future studies investigating atVNS in long COVID, we recommend to minimize the effect of placebo in clinical trials by using double-blind placebo (sham)-controlled designs in order to be sure that the potential effect found will be reliably attributable to atVNS. In sum, by improving vagal signaling and by enhancing GABA and NA, which are synthesized by the same nuclei in the brainstem that are impacted by long COVID, atVNS may have the potential to accelerate the affective and cognitive recovery in patients suffering from long COVID. We base our conclusion on previous evidence suggesting that a) this intervention has been shown to ameliorate depressive symptoms [35] and that b) the same cognitive processes promoted by atVNS [18,36] are impacted by long COVID [37].

## 6. AtVNS as Adjuvant Treatment to Pharmacotherapy?

At this point, there are no accepted medications that have been identified to reduce long COVID symptoms. As pointed out by Jarrott [1], the development of a pharmaceutical agent from the discovery of a good candidate to its approval for use typically takes 10 years or more. Given the high demands by long COVID patients for pharmacological treatment, it is currently accepted to follow the so-called “treating the host” strategy using generic drugs. To date, to evaluate pharmaceutical agents for long COVID, at least 26 randomized trials [5] are in progress examining anti-inflammatory, antihistamine, antidepressant, anticlotting, and steroid medication. Anti-inflammatory drugs, such as colchicine, might be useful, given that in long COVID increased proinflammatory cytokines have been associated with chronic inflammation [5]. Antihistamines, such as famotidine and loratadine, might be helpful considering that long COVID has been associated with T cell perturbations, which are known to be regulated by histamine [114]. Antidepressants, such as vortioxetine, might be useful because they are known to modulate the cellular and cytokine systems and to ameliorate mood and anxiety in addition to regulating sleep and circadian rhythms often impacted by long COVID [115]. Furthermore, anticlotting medication, such as rivaroxaban, might be effective in contrasting vascular inflammation and coagulation abnormalities, which arise in many long COVID patients [116]. Finally, steroid medication, such as deflazacort, might be useful in ameliorating shortness of breath, a typical symptom of patients suffering from long COVID [117].

Unfortunately, pharmacotherapy is subjected to the well-known problem of non-responders. That is, a lack of (or poor) response to medications presupposes not only an aggravation or protraction of the symptoms but also longer hospital care and longer withdrawal from social and working life [118]. Hence, until specific pharmaceutical agents to treat long COVID will be available, it is important to develop potential novel non-pharmacological interventions to treat long COVID [6]. Furthermore, long COVID has been considered a complex condition likely to be triggered by multiple causes [5], which is likely to require and benefit from an approach encompassing multiple treatments. Accordingly, in order to avoid harmful drug–drug interactions, non-pharmacological ADD-ON tools to current pharmacological treatments might be particularly useful. Finally, the pharmaceutical agents for long COVID proposed so far target neurological outcomes, such as shortness of breath, disturbances of sleep and circadian rhythms, fatigue, headache, depression, and anxiety, but not cognitive outcomes, such as deficits in attention, memory, and cognitive control. To deal with this matter, new adjuvant techniques that can be used in conjunction with current pharmacological intervention strategies are needed in order to develop successful long-COVID treatments to improve mood and to compensate for the cognitive failures caused by this post-viral disorder at the same time. Compared to other non-invasive brain stimulation techniques, such as transcranial magnetic stimulation (TMS) and transcranial direct current stimulation (tDCS), which are also known to enhance cognition [119], atVNS is ideal because it is the only one to directly enhance vagal signaling—which in turn is dysfunctional in long COVID [15,16]. In the following, we will delineate how atVNS may act as such an ADD-ON, outlining the efficiency and practicality of atVNS with respect to treating long COVID.

## 7. AtVNS Safety and Feasibility in Real-World Settings

As pointed out in Section 5, atVNS seems to be an effective tool in boosting mood and processes related to attention, memory, and cognitive control [35,36,120,121], all of which are deficient in long COVID [37,63]. AtVNS is safe and well tolerated and has been linked to only minor adverse effects, such as a tickling, heating, or itchy feeling under the electrodes [122]. However, it is important to keep in mind that the use of atVNS should be avoided in the case of pregnancy, head trauma, metal object in the body, such as cardiac pacemaker or active implantable medical devices, and wounded skin. With regard to feasibility, atVNS can be employed as an ecological momentary intervention (EMI), given that it is compact and portable and does not need to be set up by medical personnel or involve any type of surgery [105]. New generations of atVNS devices, such as tVNS^®^ L, are easy to use via a friendly mobile app and the earphone-like electrodes are comfortable to wear in day-to-day life, similar to regular in-ear headphones. Hence, the safety, tolerability, and feasibility profile of atVNS makes of this method an ideal tool to treat long COVID compared to other non-invasive brain stimulation techniques, such as TMS and, to a lesser extent, tDCS, which are restricted to clinical settings and can only be applied by trained specialists. Indeed, the possibility to apply atVNS at home is particularly appealing for long COVID for two reasons. First, given that patients are often too tired to leave home, atVNS will provide them accessibility and continuity of treatment. Second, given that atVNS is contactless, it can be applied by the patients themselves. This will assure extra safety given that new data suggests that compared to individuals with a first infection, reinfection can cause an exacerbation of long COVID [4]. Importantly, atVNS could also be combined at home with cognitive training video games, such as “Neuroracer” [123], to boost potential long-term effects of atVNS in long COVID patients. In this respect, it would be important to carry out the atVNS stimulation overlapping with the cognitive training. This might be an important factor given that an intervention study over 25 weeks moderate vagus nerve stimulation intensities were delivered during the memory consolidation period, and this did enhance recognition memory [112]. Similarly to other brain stimulation techniques, such as tDCS, it may thus be that successful enhancement requires stimulation during cognitive training [124] in order to achieve the consolidation of long-term learning. In sum, atVNS is a safe, feasible, and simple-to-use tool, which makes it the perfect EMI to improve affective and cognitive outcomes in long COVID.

## 8. Conclusions

Long COVID has been considered a complex condition likely to be triggered by multiple causes, which might likely require and benefit from an approach encompassing multiple treatments. Two of these causes might be persistent brainstem dysfunction and disrupted vagal signaling. So far, the pharmaceutical agents proposed for long COVID target neurological, but not affective and cognitive outcomes at the same time. The current position paper proposes the potential application of atVNS as an ADD-ON instrument to enhance cognition and ameliorate affective symptoms in long COVID. Compared to other non-invasive brain stimulation methods, this innovative technique (**a**) boosts vagal signaling by directly activating the nuclei in the brainstem (i.e., NST and LC), which are known to be hypoactive in individuals suffering from long COVID, (**b**) ameliorates affective symptoms, and (**c**) enhances attention, memory, and cognitive control, the same processes impacted by long COVID. If this approach will be successful in treating long COVID, it will prove the causal involvement of a disrupted vagal signaling in this condition. Given that atVNS is a non-pharmacological intervention, its ADD-ON to standard pharmaceutical agents will be helpful for non-responders and will prevent harmful drug–drug interactions, making of this method an ideal tool to compensate for the cognitive failures caused by this condition. Furthermore, considering that the earphone-like electrodes are comfortable to wear in day-to-day life similar to regular in-ear headphones, atVNS has the potential to turn out to be an optimal EMI to boost affective and cognitive outcomes in long COVID. In view of the fact that atVNS is contactless and can be applied at home by the patients themselves without the help of trained specialists, this method will assure accessibility and continuity of treatment without any risk of reinfection that might exacerbate long COVID.

## Data Availability

Not applicable.
